# RANKL-RANK signaling regulates osteoblast differentiation and bone formation

**DOI:** 10.1038/s41413-018-0040-9

**Published:** 2018-11-27

**Authors:** Xu Cao

**Affiliations:** Department of Orthopedic Surgery, Johns Hopkins University School of Medicine, Ross Building, Room 229, 720 Rutland Ave, Baltimore, MD 21205 USA

In the recent two decades, it has been well elucidated that receptor activator of nuclear factor-κB ligand (RANKL; also known as TNFSF11) binding to its receptor RANK (also known as TNFRSF11A) drives osteoclast development as the crucial signaling pathway.^1–3^ However, accumulating evidence also implies that osteoblastic RANKL regulates osteoblastogenesis.^4–6^ The studies “RANKL signaling in bone marrow mesenchymal stem cells negatively regulates osteoblastic bone formation” by Chen et al. in the current issue of *Bone Research*, and “Coupling of bone resorption and formation by RANKL reverse signalling” by Yuki Ikeuchi et al. in *Nature* (2018;561:195–200) reveal that RANKL-RANK signaling regulates osteoblastogenesis in addition to its role in osteoclastogenesis.

**Fig. 1:**
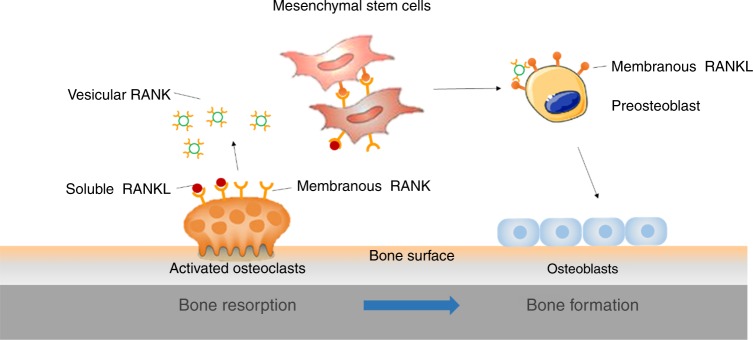
Mechanism of RANKL signaling in osteoblast differentiation. RANKL signaling drives osteoclastogenesis. In BMSCs, RANKL binding to RANK activates RANKL forward signaling, which inhibits osteoblast differentiation. Maturing osteoclasts secrete vesicular RANK which activates RANKL reverse signaling in osteoblasts and promotes osteoblast differentiation. During osteoblastogenesis, the RANK expression is reduced and RANKL forward signaling on osteoblast differentiation is relieved

Chen et al. demonstrate that RANK is expressed in bone marrow mesenchymal stem cells (BMSCs) and is decreased during osteogenic differentiation. RANK silencing significantly promotes, while overexpression suppresses, the osteoblast differentiation of BMSCs in vitro. Mice with a conditional knock-out of RANK in MSCs (Prx1-Cre: RANK^flox/flox^) show a significant increase of osteoblast differentiation and bone formation. Interestingly, in an ovariectomized mouse model, RANK conditional knock-out mice exhibit resistance to ovariectomy-induced bone loss relative to the sham-operated mice. This study reveals that RANKL forward signaling in BMSCs functions as a negative regulator in osteoblast differentiation and bone formation (Fig. 1).

RANKL belongs to the tumor necrosis factor family and its bidirectional signaling has been indicated.^6,7^ The most recent study by Yuki Ikeuchi et al. provided evidence for RANKL reverse signaling in the coupling of bone resorption and formation.^8^ RANK in small extracellular vesicles, secreted from the maturing osteoclasts, binds osteoblastic RANKL and promotes osteoblast differentiation by triggering RANKL reverse signaling, which activates runt-related transcription factor 2 (Fig. 1). In vivo, the authors also establish a mouse model (RANKL^P29A^) to inhibit RANKL reverse signaling but not forward signaling. Bone formation is disrupted in RANKL^P29A^ mice compared with wild-type mice after recombinant RANKL is administered. At last, the authors show that targeting RANKL reverse signaling prevents decreased bone formation by compensating for the shortage of coupling signals. The results suggest that RANKL reverse signaling is involved in the bone formation as a potential pharmacological target.

These two studies convincingly demonstrate the functions of RANKL-RANK forward and reverse signaling in the regulation of osteoblast differentiation and bone formation. Chen et al. show that RANKL binds to RANK and inhibits osteoblastogenesis, while Yuki et al. demonstrate that vesicular RANK from maturing osteoclasts promotes osteoblastogenesis for the bone formation through RANKL reverse signaling. Specifically, RANKL forward signaling activates NF-κB for degradation of β-catenin. In RANKL reverse signaling, a proline-rich motif in the RANKL cytoplasmic tail interacts with Src homology 3 domains and activates PI3K.

In summary, these two reports provide evidence that RANKL-RANK forward and reverse signaling regulates osteoblast differentiation and bone formation. The forward signaling inhibits osteogenic differentiation and the reverse signaling promotes osteoblast differentiation for bone formation (Fig. 1). Not only do the findings discover the novel regulatory roles of RANKL-RANK signaling in osteoblastogenesis but also they provide a potential pharmacological target in an anabolic therapy.
